# Peiminine inhibits colorectal cancer cell proliferation by inducing apoptosis and autophagy and modulating key metabolic pathways

**DOI:** 10.18632/oncotarget.17411

**Published:** 2017-04-25

**Authors:** Zhi Zheng, Liting Xu, Shuofeng Zhang, Wuping Li, Fangfang Tou, Qinsi He, Jun Rao, Qiang Shen

**Affiliations:** ^1^ Department of Internal Medicine 5th Division, Jiangxi Provincial Key Laboratory of Translational Medicine and Oncology, Jiangxi Cancer Hospital, Jiangxi Cancer Center, Nanchang, 330029, PR China; ^2^ School of Graduate Study, Medical College of Nanchang University, Nanchang, 330029, PR China; ^3^ Department of Clinical Cancer Prevention, The University of Texas MD Anderson Cancer Center, Houston, TX, 77030, USA; ^4^ Department of Pharmacology, Beijing University of Chinese Medicine, Beijing, 100102, PR China

**Keywords:** colorectal cancer, peiminine, natural product, metabolomics, cancer therapy

## Abstract

Peiminine, a compound extracted from the bulbs of *Fritillaria thunbergii* and traditionally used as a medication in China and other Asian countries, was reported to inhibit colorectal cancer cell proliferation and tumor growth by inducing autophagic cell death. However, its mechanism of anticancer action is not well understood, especially at the metabolic level, which was thought to primarily account for peiminine's efficacy against cancer. Using an established metabolomic profiling platform combining ultra-performance liquid chromatography/tandem mass spectrometry with gas chromatography/mass spectrometry, we identified metabolic alterations in colorectal cancer cell line HCT-116 after peiminine treatment. Among the identified 236 metabolites, the levels of 57 of them were significantly (*p* < 0.05) different between peiminine-treated and -untreated cells in which 45 metabolites were increased and the other 12 metabolites were decreased. Several of the affected metabolites, including glucose, glutamine, oleate (18:1n9), and lignocerate (24:0), may be involved in regulation of the phosphoinositide 3-kinase/Akt/mammalian target of rapamycin (mTOR) pathway and in the oxidative stress response upon peiminine exposure. Peiminine predominantly modulated the pathways responsible for metabolism of amino acids, carbohydrates, and lipids. Collectively, these results provide new insights into the mechanisms by which peiminine modulates metabolic pathways to inhibit colorectal cancer cell growth, supporting further exploration of peiminine as a potential new strategy for treating colorectal cancer.

## INTRODUCTION

Colorectal cancer (CRC) is the third most common cancer in the world and the fourth most common cause of cancer-related deaths [1, 2]. The development and progression of CRC are influenced by lifestyle, genetic, and environmental factors [3, 4]. Although the introduction of advanced surgical and systemic therapeutic options has improved the prognosis for CRC over the past few years, the current challenges still led to new effective less toxic chemotherapeutic agents in treatment of colon cancer [5, 6]. Apoptosis and autophagy are two distinct processes, coordinately regulating cell survival and cell death, and occur simultaneously in cancers [7, 8]. Type I programmed cell death (PCD), apoptosis, is a biological process with a crucial role in normal development and tissue homeostasis [9]. Autophagic cell death, also called type II programmed cell death, is another alternative PCD pathway other than apoptosis (type I cell death), which has also been validated in colorectal carcinoma [10–12]. In addition, the apoptosis and autophagy mechanisms are involved in the metabolism of CRC and play an important role in the multifactorial etiology of CRC. So focusing on the modulation of apoptosis and autophagy has enabled the identification of potential new therapeutic strategies for the treatment of colon cancer [13].

Metabolomics is a high-throughput technique for global, comparative, and semi-quantitative assessment of all metabolites in specific cells, tissues, or bodily fluids at a given time [14]. Recently, substantial evidence has supported the use of metabolomics for diagnosing cancer, predicting its recurrence, and determining prognosis [15–19]. Metabolomics has also proved useful in identifying novel cancer biomarkers and developing cancer therapeutics [15–17]. To date, researchers have used metabolomics in investigations of various cancers, including CRC, gastric cancer, pancreatic cancer, and liver cancer, using samples such as tissue, cells, serum, plasma, urine, or saliva [15–20]. For example, high-performance liquid chromatography–quadrupole time-of-flight MS has been used to profile the urine metabolites of cancer patients [18]. The metabolites involved in glycolysis and β-oxidation were differentially changed in patients, which suggested that the excellent performance and simplicity of this metabolomics-based approach could augment the current modalities used for cancer diagnosis. Non-targeted gas chromatography–time-of-flight MS metabolomic method in conjunction with random forests was also used to identify metabolic characteristics involved in hepatocellular carcinogenesis [19], which identified the metabolites closely associated with pathways involved in energy metabolism, macromolecular synthesis, and maintenance of the redox balance that protects tumor cells from oxidative stress. For CRC, other investigators have analyzed various types of clinical samples using metabolomic platforms, including nuclear magnetic resonance spectrometry, gas chromatography (GC)/MS (GC/MS), liquid chromatography/MS, and capillary electrophoresis/MS to discover novel metabolic biomarkers for CRC diagnosis and to elucidate how CRCs develop [20–23].

**Figure 1:**
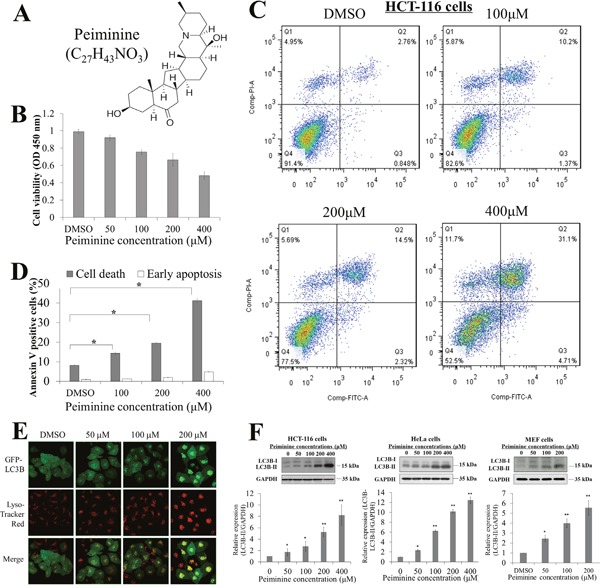
Peiminine inhibits growth and induces apoptosis in HCT-116 cells **(A)** Molecular structure and formula of peiminine. **(B)** Results of viability assay showing dose-dependent decrease in HCT-116 cell viability after peiminine treatment at 0, 50, 100, 200 and 400 μM concentrations. **(C)** Double staining of peiminine-treated and untreated HCT-116 cells with propidium iodide and annexin V and vehicle control (DMSO) for 24 hours for flow cytometry. **(D)** Percentage of dead and early-apoptotic cells in three independent experiments.* *p* ≤ 0.05. DMSO, dimethyl sulfoxide. **(E)** Confocal microscopy images of the Hela cells treated with DMSO(0), 50, 100 and 200 μM peiminine for 24 h. Green coloring indicates the presence of stably expressed LC3II. Red coloring indicates the presence of lysosomes (magnification, ×630). **(F)** Dose-dependent increases in LC3B-II/LC3B-I ratio with peiminine treatment at 0, 50, 100, 200 and 400 μM concentrations in HCT-116, HeLa, and MEF cells. Three independent immunoblot assays were performed and gray scale images of each assay were scanned and presented as a bar chart in which the vertical axis is the mean gray scale ± SD. * indicates statistical differences at p < 0.05 and ** indicates statistical differences at p < 0.01.

Peiminine (Figure 1A) is a natural compound extracted from the bulbs of *Fritillaria thunbergii* (Liliaceae family), which is widely used in traditional Chinese medicine for treating various diseases, including cancer. Our previous study reported that peiminine represses CRC cell proliferation and tumor growth by inducing autophagic cell death *in vitro* and *in vivo* [24]. However, the anticancer mechanism of peiminine is not well understood. Specifically, the metabolic alterations induced in CRC cells by peiminine-based treatment have yet to be explored. Using an established metabolomics profiling platform based on the combination of ultra-performance liquid chromatography-tandem MS (UPLC/MS/MS) with GC/MS, we for the first time metabolically profiled the CRC cell line HCT-116 before and after peiminine exposure. The results showed remarkable variations in the levels of key metabolites (such as glucose and glutamine) in core metabolic pathways relevant to the metabolism of amino acids, carbohydrates, and lipids. Our results provide new insights into the metabolic alterations associated with peiminine-based treatment in colon cancer cells and extend our understanding of peiminine's anticancer mechanisms.

## RESULTS

### Peiminine induces apoptosis and autophagy in HCT-116 cells

The molecular structure of peiminine is shown in (Figure 1A). To demonstrate and validate that peiminine induces apoptosis and autophagy, we treated HCT-116 cells with 50, 100, 200, and 400 μM of peiminine for 48 h and compared the number of viable cells. We observed that peiminine treatment decreased the number of viable HCT-116 cells in a dose-dependent manner (Figure 1B), and also significantly increased the number of annexin V-positive HCT-116 cells in a dose-dependent manner for 200 and 400 μM of peiminine, as shown by apoptosis assays in Figure 1C. This alteration was consistent with the flow cytometry assessments which demonstrated that peiminine treatment at the same concentrations induced early apoptosis in HCT-116 cells (Figure 1C & 1D). These results suggested that peiminine inhibits the growth of HCT-116 cells and induces their apoptosis. To investigate the autophagy-inducing ability of peiminine, we assessed autophagy activation potential in GFP-LC3 stable-expression HeLa cells. We applied a gradient dosage of peiminine (50, 100 and 200 μM) in HeLa-GFP-LC3 cells with a control solvent and observed an increase in the GFP (green color) and lysosomes (red color) puncta in a dose-dependent manner (Figure 1E). Immunoblot assays against LC3B also showed a significant elevation of the LC3B-II/LC3B-I protein in the 50, 100, 200 and 400 μM peiminine-treated HCT-116 (as comparison), HeLa and MEF cells, suggesting that peiminine induces autophagy in colon cancer cells as well as other cells (Figure 1F).

### Metabolic profiling of HCT-116 cells

To comprehensively understand the metabolic changes induced by peiminine treatment in HCT-116 cells, we employed a well-established global metabolic profiling approach that combined GC/MS with UPLC/MS/MS and identified a total of 236 metabolites in HCT-116 cells, which were mapped to 8 super-pathways and 55 sub-pathways (Supplementary Table 1). The identified 236 metabolites included 60 amino acids, 25 carbohydrates, 15 co-factors or vitamins, 81 lipids, 24 nucleotides, 16 peptides, 7 xenobiotics, and 8 metabolites involved in energy metabolism (Supplementary Table 1).

### Metabolic variation in HCT-116 cells after peiminine exposure

**Figure 2:**
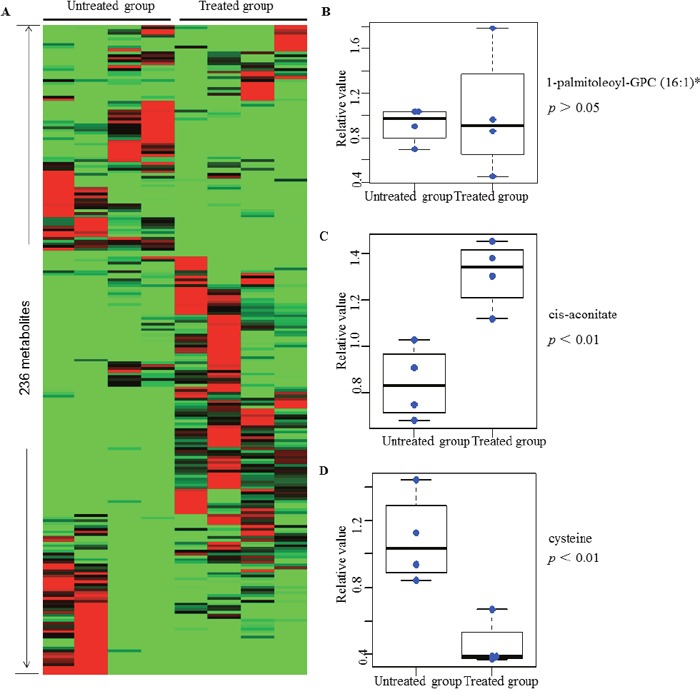
Heat map representation of 236 metabolites between treated and untreated group in clustering analysis Each line represents a metabolite. The deeper the red color, the higher its content in the cell lines; similarly, the deeper the green color, the lower its content in the cell lines. Three metabolites mentioned in the text are shown in greater detail by boxplot. The *p* values were calculated between the peiminine-treated and untreated groups. List of 236 metabolites is provided in Supplementary Table 1.

To determine the metabolic similarities and differences among control and peiminine-treated HCT-116 cells, we subjected the metabolic profiles of all samples to hierarchical cluster analysis. As shown in Figure 2A, the four samples in the treatment group clustered together, as did the four samples in the untreated group, indicating that the metabolite profiles discriminated the peiminine-treated group from the untreated group. Moreover, apart from certain metabolites such as 1-palmitoleoyl-GPC (16:1)* displayed more or less stable (Figure 2B), most of 236 identified metabolites differed significantly between the treated and untreated groups. In most cases, peiminine reduced the levels of metabolites that were highly abundant in untreated HCT-116 cells and increased the levels of metabolites that were found in low levels in untreated cells. For example, cis-aconitate level in the peiminine-treated group was higher than those in the untreated group (Figure 2C), but cysteine was more abundant in the untreated group than in the peiminine-treated group (Figure 2D).

**Figure 3:**
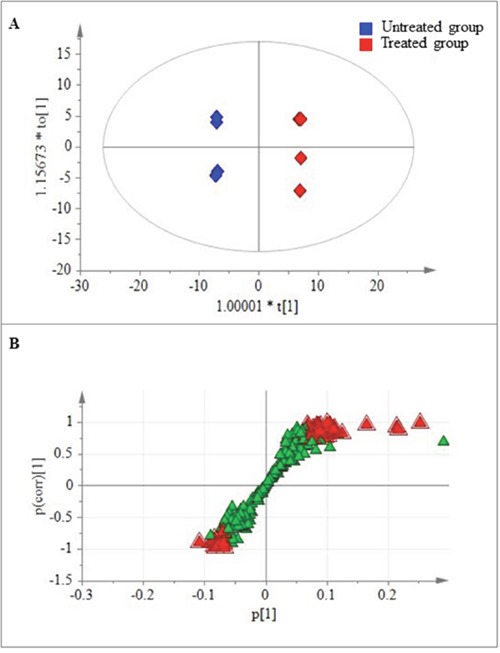
Metabolites found at significantly different levels in treated and untreated HCT-116 cells **(A)** Plot of the scores from the PLS-DA model of the treated and untreated groups. **(B)** Loading plot from the PLS-DA model of the treated and untreated groups. The boxes represent the tested samples while metabolites are displayed as triangles. Metabolites playing key roles for separation are marked with red triangle.

To further understand the metabolic changes associated with peiminine exposure in HCT-116 cells, we employed PLS-DA to identify the metabolites that separated the treated and untreated groups. The analysis showed that the levels of 57 metabolites (Table 1; Figures 3A, 3B) were significantly different in the two groups (*p ≤* 0.05). Of these metabolites, 45 increased and 12 decreased, with the magnitude of the changes ranging from 0.14- to 10.72-fold. Particularly among the 45 increased metabolites, glutamine, glucose, and glycerol were included, which are key molecules involved in the metabolism of amino acids, carbohydrates, and lipids, respectively. The remaining 12 decreased metabolites included four amino acids, one co-factor & vitamin, four lipids, and three nucleotides.

**Table 1:** Metabolites found at significantly different levels in peiminine-treated and untreated HCT-116 colorectal cancer cells

Super-pathway	Biochemical Identifier	Fold Change*	P value
Amino acid	Glutamine	2.68	1.24×10^-2^
	Ophthalmate	10.72	9.59×10^-6^
	Cadaverine	0.14	3.31×10^-3^
	Cysteine	0.42	5.63×10^-3^
	SAH	0.55	9.13×10^-6^
	Taurine	2.88	1.61×10^-3^
	Putrescine	0.18	2.25×10^-3^
	Urea	2.60	3.16×10^-6^
Carbohydrate	Glucose	3.67	1.14×10^-2^
	Glycerate	2.01	4.17×10^-2^
	UDP-N-acetylglucosamine/galactosamine	2.03	4.85×10^-3^
Co-factors and vitamins	Thiamin (vitamin B1)	0.53	4.10×10^-2^
Energy	Acetylphosphate	1.72	4.44×10^-3^
	Fumarate	1.60	4.70×10^-3^
	Malate	1.64	2.00×10^-2^
Lipid	Carnitine	0.34	2.61×10^-4^
	Propionylcarnitine (C3)	0.27	1.46×10^-6^
	Acetylcarnitine (C2)	0.30	1.10×10^-2^
	17-methylstearate	2.58	2.51×10^-3^
	2-hydroxyglutarate	1.91	3.21×10^-3^
	Glycerol	1.81	3.24×10^-3^
	Myo-inositol	1.96	4.28×10^-3^
	10-heptadecenoate (17:1n7)	1.90	3.79×10^-2^
	13-octadecenoate (18:1n5)	2.04	1.12×10^-2^
	Lignocerate (24:0)	3.38	2.63×10^-2^
	Myristate (14:0)	2.00	1.83×10^-3^
	Nonadecanoate (19:0)	1.98	1.03×10^-2^
	Oleate (18:1n9)	2.07	5.15×10^-4^
	1-arachidonoyl-GPI (20:4)*	3.30	2.02×10^-2^
	1-oleoyl-GPE (18:1)	2.10	1.32×10^-3^
	1-oleoyl-GPI (18:1)*	2.50	2.66×10^-2^
	1-oleoyl-GPS (18:1)	3.51	4.35×10^-2^
	1-stearoyl-GPC (18:0)	2.55	1.47×10^-2^
	1-stearoyl-GPE (18:0)	3.04	1.09×10^-3^
	1-stearoyl-GPS (18:0)*	2.46	6.16×10^-4^
	1-stearoylglycerol (18:0)	2.16	4.13×10^-2^
	Choline phosphate	0.34	2.74×10^-2^
	Phosphoethanolamine	6.41	2.29×10^-4^
	Dihomo-linolenate (20:3n3 or n6)	3.51	1.97×10^-2^
	Docosahexaenoate (22:6n3)	1.94	1.57×10^-2^
	Docosapentaenoate (n3 22:5n3)	2.79	1.39×10^-2^
	Eicosapentaenoate (20:5n3)	2.56	1.11×10^-2^
	Linoleate (18:2n6)	2.42	1.10×10^-2^
	Linolenate [alpha or gamma; (18:3n3 or 6)]	2.66	5.71×10^-3^
	Valerate	3.59	3.94×10^-3^
	Sphinganine	2.22	3.23×10^-2^
	Sphingosine	1.97	1.25×10^-3^
Nucleotide	Adenine	0.59	7.68×10^-4^
	Pseudouridine	0.35	6.26×10^-3^
	Xanthine	0.51	1.44×10^-3^
Peptide	Alanylleucine	2.26	2.83×10^-2^
	Phenylalanylalanine	2.73	2.09×10^-3^
	Gamma-glutamylglutamine	3.36	2.75×10^-3^
	Gamma-glutamylisoleucine*	4.40	1.10×10^-2^
	Gamma-glutamylleucine	10.11	1.23×10^-3^
	Gamma-glutamylthreonine*	9.06	4.08×10^-3^
Xenobiotic	Erythritol	1.86	2.15×10^-4^

* represents fold change between treated and untreated. GPC: glycerophosphocholine; GPE: glycerophosphoethanolamine; GPI: glycerophosphoinositol; GPS: glycerophosphatidylserine; SAH: S-adenosylhomocysteine; GSH: glutathione, reduced; GSSG: glutathione, oxidized; DHA: docosahexaenoate; DPA: docosapentaenoate; EPA: eicosapentaenoate.

### Altered metabolic pathways in peiminine-treated cells

#### Amino acid metabolism

**Figure 4:**
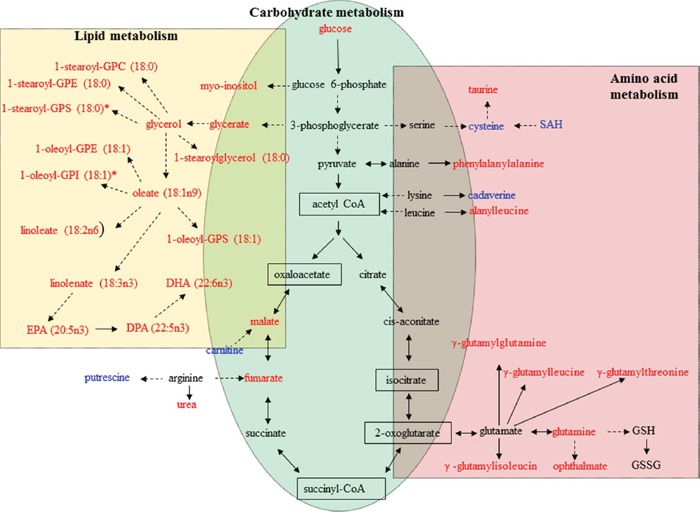
Metabolic pathways involving metabolites that were significantly increased or decreased in peiminine-treated HCT-116 cells Metabolites undetected in both the treated and untreated groups are shown in the black boxes. Metabolites in red or blue exhibited increased or decreased levels, respectively, in the peiminine-treated group. GPC: glycerophosphocholine; GPE: glycerophosphoethanolamine; GPI: glycerophosphoinositol; GPS: glycerophosphatidylserine; SAH: S-adenosylhomocysteine; GSH: glutathione, reduced; GSSG: glutathione, oxidized; DHA: docosahexaenoate; DPA: docosapentaenoate; EPA: eicosapentaenoate.

The metabolism of amino acids has several important roles in multiple aspects of cell biology, in addition to amino acids’ well-known role as building blocks in protein synthesis [29]. Individual amino acids may play specific roles in metabolic pathways. For example, glutamate, which plays critical roles in protein structure, nutrition, metabolism, and signaling, is one of the most abundant of the amino acids [30]. In addition, glutamine and aspartate are necessary for *de novo* purine and pyrimidine synthesis. Other amino acids, including tryptophan and arginine, play an important role in supporting cellular proliferation and anabolic growth. As shown in Figure 4, levels of 8 metabolites involved in amino acid metabolism, including glutamine, cysteine, and S-adenosylhomocysteine (SAH), were significantly changed (4 upregulated and 4 downregulated, *p ≤* 0.05) in peiminine-treated HCT-116 cells. For example, the level of SAH decreased, but the concentration of glutamate did not change, perhaps because of fewer live cancer cells after peiminine treatment [35]. In contrast, the levels of other relevant metabolites, including glutamine and four peptides (γ-glutamylglutamine, γ-glutamylisoleucine, γ-glutamylleucine, and γ-glutamylthreonine) were significantly increased (*p ≤* 0.05) in HCT-116 cells treated with peiminine. These results may indicate that peiminine-treated cells exhibit increased amino acid metabolism activity, which plays vital roles in cellular proliferation [30]. Additionally, levels of ophthalmate, an analog of glutathione, increased more than those of any other identified metabolite, suggesting that peiminine treatment induces apoptosis in HCT-116 cells by causing glutathione depletion or oxidative stress [31].

#### Carbohydrate metabolism

Carbohydrate metabolism, which includes glycolysis and the tricarboxylic acid cycle, generates key products that promote cell survival and growth. In particular, glycolysis plays a dominant and essential role in the metabolism of rapidly proliferating cells. We found that the levels of all 3 identified metabolites involved in glycolysis and the tricarboxylic acid cycle (glucose, malate and fumarate) were significantly higher in peiminine-treated HCT-116 cells than in untreated cells. As shown in Figure 4, the elevated level of glucose found in peiminine-treated HCT-116 cells may indicate increased aerobic glycolysis in apoptotic cancer cells [32]. These results suggest that peiminine promotes glycolysis to induce apoptosis in HCT-116 cells.

#### Lipid metabolism

Lipids form a diverse group of water-insoluble molecules, including triacylglycerides, phosphoglycerides, sterols, and sphingolipids, which play several important roles at the cellular and organismal levels [33]. Cancer cells rely on lipids as cellular building blocks for membrane formation, energy storage, and the production of signaling molecules. As shown in Table 1 and Figure 4, levels of 32 lipids were significantly altered after peiminine treatment in colon cancer cells. Levels of 16 metabolites involved in lipid metabolism were increased in HCT-116 cells after peiminine treatment, including several essential polyunsaturated fatty acids, such as docosahexaenoate (ω-3) and linoleate (ω-6). Moreover, levels of a variety of lysolipids, such as 1-stearoyl-GPC (glycerophosphocholine), 1-stearoyl-GPE (glycerophosphoethanolamine), 1-oleoyl-GPE, 1-stearoyl-GPI (glycerophosphoinositol), 1-arachidonoyl-GPI, and 1-oleoyl-GPS (glycerophosphatidylserine), were higher in peiminine-treated HCT-116 cells than in untreated cells.

## DISCUSSION

To fully understand the mechanisms of peiminine's antitumor action, here we for the first time performed metabolomic analysis for HCT-116 cells treated with peiminine. Taking advantage of an established non-targeted metabolomic profiling platform that combined UPLC/MS/MS with GC/MS, we identified 236 metabolites in HCT-116 cells, the broadest metabolome of HCT-116 cells identified to date [27–28]. Moreover, these 236 metabolites were mapped to 8 super-pathways and 55 sub-pathways. Notably, most of the central metabolic pathways were included among the identified metabolites, demonstrating that this non-targeted metabolomic profiling platform successfully elucidated the metabolome in HCT-116 cells. More importantly, we uncovered remarkable metabolic changes in HCT-116 cells after peiminine treatment, including changes in the levels of several important metabolites and metabolic pathways involved in amino acid, carbohydrate, and lipid metabolism. These metabolic changes in response to peiminine may be related to apoptosis in CRC cells with peiminine exposure. Our results thus provide new insights into the metabolic pathways affected by peiminine and establish a foundation for further improvement and application of peiminine in cancer treatment.

We found that treatment with peiminine significantly promoted apoptosis in CRC cells. Similarly, our previous study revealed that peiminine induces cell death and enhances autophagic flux in HCT-116 cells [24]. In particular, that study demonstrated that peiminine enhances autophagic flux by dephosphorylating mammalian target of rapamycin (mTOR) through the phosphoinositide 3-kinase (PI3K)/Akt)/mTOR and AMP-activated protein kinase pathways. The PI3K/Akt/mTOR pathway is a master regulator of aerobic glycolysis and cellular biosynthesis. When this pathway is activated in tumor cells, many of the metabolic activities that support cellular biosynthesis are enhanced, including uptake of glucose, amino acid metabolism, lipid synthesis, and uptake of other nutrients [34–35]. Peiminine treatment elevated the levels of 45 of 57 significantly changed metabolites, including glucose, glutamine, oleate (18:1n9), and lignocerate (24:0), increased in treated HCT-116 cells. In particular, the markedly higher levels of amino acids in treated cells than in untreated cells likely stimulated the mTOR pathway, and increased glutamine levels to stimulated mTOR activity, which validated that peiminine treatment activates PI3K/Akt/mTOR pathway, in consistence with the findings we reported in the other studies [36, 49].

Regulation of oxidative stress plays an important role in both tumor development and responses to anticancer therapies. Under normal physiological conditions, the generation of reactive oxygen species is balanced by oxidative stress response mechanisms. When this balance is disturbed by excessive reactive oxygen species or by a deficient antioxidant defense, oxidative stress occurs, leading to enhanced cell damage. The present study found a considerable decrease in SAH levels in cells treated with peiminine, which functions as a major cellular anti-oxidant [42]. In contrast, levels of several reactive oxygen species-related metabolites, including myo-inositol and taurine, increased in peiminine-treated cells. These metabolites were also identified in a study demonstrating the therapeutic effect of silibinin in CRC cells [37]. Furthermore, we found that HCT-116 cells treated with peiminine exhibited a significant accumulation of glutamine and multiple γ-glutamyl amino acids, which may reflect changes in glutathione turnover and availability in cells treated with peiminine. These results suggest that peiminine restores impaired systemic redox homeostasis in cancer cells.

Cancer cells often undergo characteristic metabolic changes [38, 39], the most well understood of which is the Warburg effect. It is characterized by an increase in glucose uptake and lactate production accompanied by a decrease in oxidative phosphorylation [40]. Increased glutamine metabolism is another commonly observed metabolic alteration in cancer cells. Glutamine metabolism supports cell growth and proliferation and plays important roles in the maintenance of cellular redox homeostasis and in cell signaling [41]. In this study, levels of both glucose and glutamine were markedly higher in peiminine-treated HCT-116 cells than in untreated cells, probably because of the increased rates of apoptosis in treated cells. Furthermore, levels of several metabolites involved in energy and glutamine metabolism, including fumarate, malate, and multiple γ-glutamyl amino acids, were also higher in peiminine-treated HCT-116 cells.

Additionally, the observed decrease in SAH levels in peiminine-treated cells may have played a key role in supporting CRC cell proliferation. SAH is known to be a potent inhibitor of S-adenosylmethionine–mediated reactions, which are important in normal cell function and survival [38]. Previous studies demonstrate that accumulation of SAH in body fluids is associated with vascular disease and tissue damage [43, 44]. Especially, SAH was a functional biomarker related to folate status in colorectal cancer [45–48, 50].

Besides amino acid and carbohydrate metabolism, lipid metabolism is also important for cancer cells. Lipids serve as cellular building blocks for membrane formation, energy storage, and the production of signaling molecules. We identified high levels of lipids in peiminine-treated HCT-116 cells. For example, one of the most significant changes we observed was the accumulation of several essential polyunsaturated fatty acids in cells treated with peiminine (Table 1; Figure 4). The essential fatty acids docosahexaenoate (ω-3) and linoleate (ω-6) are vital precursors for the generation of lipid mediators, such as eicosanoids and leukotrienes, which regulate numerous cellular functions including inflammation [51, 52]. In general, ω-3–derived metabolites exert anti-inflammatory functions, whereas ω-6–derived metabolites exert proinflammatory functions. The accumulation of polyunsaturated fatty acids may reflect CRC cells’ increasing need for the generation of lipid mediators in response to the exposure of peiminine.

Moreover, levels of a variety of lysolipids (similar to phospholipids in that they are composed of a glycerol backbone and head group but containing only one single fatty acyl chain), such as 1-stearoyl-GPC, 1-stearoyl-GPE, 1-oleoyl-GPE, 1-stearoyl-GPI, 1-arachidonoyl-GPI, and 1-oleoyl-GPS, were increased in HCT-116 cells after peiminine treatment. The observed abundance of lysolipids in peiminine-treated cells suggests the involvement of a phospholipase enzyme, phospholipase, in hydrolyzing membrane phospholipids to release free fatty acids from the phospholipids. Many phospholipase A2 isoforms are associated with inflammation because of their ability to release arachidonic acid, which generates inflammatory eicosanoids via the lipoxygenase and cyclooxygenase pathways [53].

Taken together, our results suggest that treatment with peiminine can induce cancer cell apoptosis and autophagy via modulating the production of metabolites, which are supportive of carcinoma cell proliferation. This study is the first to reveal the metabolic alterations in HCT-116 CRC cells treated with peiminine. The remarkable metabolic variations upon peiminine exposure include differential metabolite production and activation of metabolic pathways related to the regulation of PI3K/Akt/mTOR pathway and oxidative stress. In parallel studies, we are currently exploring the effect of peiminine exposure on proteomics and transcriptomics in cancer cells to further elucidate the mechanisms of action of peiminine. Moreover, *in vivo* studies will determine the potential usefulness of peiminine for treating CRC and other cancers. Future research should explore potential interacting targets of peiminine and determine its precise mechanisms of action. Such studies could employ structure- and function-based *in silico* analyses, peiminine-based tool compounds, and other methods to characterize the direct and indirect targets of peiminine. Because peiminine has a low toxicity profile and relatively moderate potency, future work should also aim to develop peiminine derivatives that improve the drug's potency without compromising its safety profile.

## MATERIALS AND METHODS

### Cell culture

HCT-116, HeLa, and mouse embryonic fibroblast (MEF) cells were obtained from ATCC (Manassas, VA). The cells were cultured in Dulbecco's modified Eagle's medium supplemented with 10% fetal bovine serum in a humidified incubator at 37°C in an atmosphere of 5% CO_2_. The cells were subcultured when they reached 90% confluence.

### Cell viability assay

HCT-116 cells were seeded in a 96-well plate (2000 cells/well) in Dulbecco's modified Eagle's medium supplemented with 2% fetal bovine serum. Peiminine solution (catalog #110751-201110; National Institutes for Food and Drug Control, Beijing, P.R. China) was applied to the cells at final concentrations of 0, 100, 200, and 400 μM. To determine cell viability, 48 h after treatment, 10 μL of the ready-to-use reagent CCK-8 (catalog # DB884; Dojindo, Kumamoto, Japan) was added to each well. The cells were then incubated for approximately 30 min at 37°C in a humidified, 5% CO_2_ atmosphere. Within 1 h of the completion of the incubation period, the absorbance of the formazan dye produced was measured at a wavelength of 450 nm using a microplate spectrometer. The measured absorbance correlated directly to the number of viable cells.

### Confocal microscopy

GFP-LC3B stable expression HeLa cells were seeded and cultured in a flask overnight. A gradient concentration of peiminine was added to the cells with final concentrations of 0, 50, 100 and 200 μM. Cells were observed with an Olympus FV1000 (Olympus) confocal microscope 24 h after treatment.

### LysoTracker red labeling

GFP-LC3B stable expression HeLa cells were treated with final concentrations of 0, 50, 100 and 200 μM peiminine for 24 h followed by incubation with 50 nM LysoTracker Red DND-99 for 2 to 3 h at 37°C. Next, LysoTracker Red was removed and cells were washed three times in PBS, incubated with fresh culture medium for 20 min. The cells were then analyzed using an Olympus FV1000 (Olympus) confocal microscope.

### Flow cytometry

To identify apoptotic cells, HCT-116 cells were treated with 200 μM peiminine or dimethyl sulfoxide as a control and cultured in Dulbecco's modified Eagle's medium supplemented with 10% fetal bovine serum for 24 h. The cells were collected and stained with propidium iodide and annexin V (BD Biosciences, Franklin Lakes, NJ) according to the manufacturer's guidelines and analyzed using a BD Influx flow cytometer (BD Biosciences).

### Metabolite profiling

Metabolite profiling of both peiminine-treated and untreated HCT-116 cells was performed as described previously using a global unbiased platform, which was a combination of three independent analytical platforms: UPLC/MS/MS optimized for basic species, UPLC/MS/MS optimized for acidic species, and GC/MS [25, 26]. Briefly, samples were prepared using the automated MicroLab STAR^®^ system from Hamilton Company. A recovery standard was added prior to the first step in the extraction process for QC purposes. To remove proteins, dissociate small molecules bound to proteins or trapped in the precipitated protein matrix, and recover chemically diverse metabolites, proteins were precipitated with methanol under vigorous shaking for 2 min (Glen Mills GenoGrinder 2000) followed by centrifugation. The resulting extract was divided into five fractions: one for analysis by GC-MS, one for analysis by UPLC-MS/MS with positive ion mode electrospray ionization, one for analysis by UPLC-MS/MS with negative ion mode electrospray ionization, one for LC polar platform, and one sample was reserved for backup. Samples were placed briefly on a TurboVap^®^ (Zymark) to remove the organic solvent. For GC, each sample was dried under vacuum overnight before preparation for analysis. For LC, the samples were stored overnight under nitrogen before preparation for analysis.

For the GC/MS analysis, the samples were re-dried under vacuum for 24 h before derivatization in the presence of liquid nitrogen using *N, O*-bis (trimethylsilyl)trifluoroacetamide. The GC column was composed of 5% phenyl, and the temperature ramp ranged from 40°C to 300°C over a 16-min span. A Finnigan Trace DSQ fast-scanning single quadrupole mass spectrometer (Thermo Fisher Scientific, Waltham, MA) using electron impact ionization was employed to measure the retention time and molecular weight (m/z) for all detectable ions.

For the UPLC/MS/MS analysis, instrument internal standards were employed to monitor instrument performance. UHPLC/MS was performed using a Waters ACQUITY UPLC system (Waters, Milford, MA) coupled to a Thermo Fisher Scientific Orbitrap Elite high-resolution mass spectrometer. Each sample was analyzed using separate dedicated columns: one optimized for positive ions and one for negative ions. The mobile phase for positive ion analysis consisted of 0.1% formic acid (FA) in H_2_O (solvent A) and 0.1% FA in methanol (solvent B), while in negative ion mode, 6.5 mM ammonium bicarbonate, pH 8.0 (solvent A) and 6.5 mM ammonium bicarbonate in methanol (solvent B) were used. Moreover, gradient was eluted directly into the mass spectrometer from 0% B to 98% B over 11 min at a flow rate of 350 μL/min. Likewise, the MS analysis recorded the retention time, molecular weight (m/z), and tandem mass spectrometry (MS/MS2) spectra of all detectable ions in the samples, which alternated between MS (99–1000 m/z) and data-dependent MS2 scans using dynamic exclusion.

The metabolites present in the treated and untreated HCT-116 cells were identified using an automated comparison with Metabolon's reference library entries. For each platform, a reference library of metabolites has already been established using approximately 1,500 authentic standards that had been analyzed in multiple concentrations and under the same conditions as the experimental samples. Each library included retention time, molecular weight (m/z), preferred adducts, and in-source fragments for all standards as well as their associated MS/MS^2^ spectra. The combination of the chromatographic retention index and MS/MS^2^ spectra signatures was used to indicate a match to a specific metabolite. Library matching for each metabolite was conducted for each sample and corrected manually if necessary. Additionally, a series of quality control and curation procedures was performed to ensure the highest data quality for the metabolomic data analysis.

### Data analysis

For the apoptosis analysis, each cell sample was analyzed in three replicates. Data are presented as means ± standard deviations. A Welch independent sample *t* test was performed to identify significant differences in apoptosis rates between the treated and untreated groups. Statistical analyses were performed using SPSS software (version 17.0; SPSS Institute, Chicago, IL). *P* values of up to 0.05 were considered statistically significant.

The metabolomic data were analyzed as described previously [26]. Briefly, data normalization was performed, and the missing values for each metabolite were inputted with the detected minimum value. All the metabolites were mapped to super-pathways and sub-pathways according to the Kyoto Encyclopedia of Genes and Genomes database [26]. Hierarchical clustering of metabolites was performed using the Pearson correlation coefficient by MultiExperiment Viewer software (version 4.8; http://www.jcvi.org/cms/research/software/). SIMCA-P software (version 13.0; MKS Data Analytics Solutions, Malmö, Sweden) was used to conduct partial least squares discriminant analysis (PLS-DA) to prevent repeat of variables, and “Par” was selected for data scaling. In the PLS-DA model, variables (metabolites) with differing importance in the project values greater than 1 were selected for independent *t*-tests. Variables with significant differences (*p ≤* 0.05) in the *t*-test results were considered to be significantly changed metabolites that separated the treated and untreated groups.

## SUPPLEMENTARY MATERIALS TABLE




