# Supplementary material for: Peiminine inhibits colorectal cancer cell proliferation by inducing apoptosis and autophagy and modulating key metabolic pathways
Source: Oncotarget. 2017 Apr 25;8(29):47619–31. doi: 10.18632/oncotarget.17411 (PMC5564592; doi:10.18632/oncotarget.17411)
Supplement: Supplementary file 1 [file oncotarget-08-47619-s001.pdf]

Peiminine inhibits colorectal cancer cell proliferation by inducing apoptosis and autophagy and modulating key metabolic pathways

SUPPLEMENTARY TABLE

Supplementary Table 1: List of 236 detected metabolites with their some important properties including retention index (RI), mass, KEGG and HMDB entry numbers

See Supplementary File 1
